# Notes and News

**Published:** 1897-11-27

**Authors:** 


### NOTES AND NEWS.

(COLLECTED AND COMMUNICATED.)

THE buildings of the Royal Eye Infirmary, at Plymouth, have just been sold for £5,650 to the proprietor of an hotel, after keen competition. The purchase will not be completed until the new hospital is ready for occupation.

NEW rules have just been adopted at the Derby Royal Infirmary. Amongst other alterations, four honorary physicians and four honorary surgeons are to be appointed instead of three, and a clinical assistant and honorary consulting dental surgeon will further increase the staff.

THE foundation-stone of the Kempton Jubilee Hospital has been laid. This building, to be called the Victoria Hospital, will stand on a site of about three acres, presented by the Duke of Cambridge. It is hoped that a sum of £5,000 will be collected, as £3,500 has already been subscribed.

THE Marquis of Ripon recently laid the foundation-stone of an infirmary to be added to the Ripon workhouse. The building is to contain accommodation for forty patients besides that for nurses. The sum of £2,650 is quoted as the estimated cost, an expenditure which appears to us to be remarkably low.

SIR WILLIAM AGNEW has opened the convalescent home at St. Anne's on-Sea, which he has presented to the Manchester Children's Hospital. The home will contain 25 patients. At the opening ceremony Sir William Agnew announced a further donation of £500 towards the endowment of his already munificent gift.

A MEETING of the Hospitals Association will be held in the Board Room of the Westminster Hospital on Thursday, December 9th, at half-past four p m. Mr. R. D. B. Acland (chairman of the Hospital Saturday Fund), will read a paper on "The Work and Aims of the Hospital Saturday Fund," and Mr. G. A. Cross (secretary of the London Homœopathic Hospital) will read a paper on "Various Methods of Distribution." The papers will be followed by a discussion.

THE foundation-stone of a new infectious diseases hospital has been laid at Gloucester.

THE new cottage hospital at Malmesbury has been opened by the Mayoress. The hospital is a two-storied building, and contains two wards with four or five beds in each, two single wards for paying patients, an operating room, and nurses and servants' quarters; there is also a detached mortuary. The site was given by Colonel and Mrs. Leice, together with a donation of £500. The cost is estimated between £1,600 and £1,700.

ON the 15th inst. the first sod of the new Cottage Hospital, Nuttall Lane, Ramsbottom, was cut by Mrs. Aitken, of Holcombe Hall, who is defraying the whole of the cost, and presenting the building to the town. The contract has been let to Messrs. Platt and Castle, of Ramsbottom, and the work is from designs, and under the superintendence of Messrs. Haywood and Harrison, architects, of Accrington and Lytham. The cost is about £3,000.

THE committee of the *Arethusa* and *Chichester* training ships, Greenhithe, Kent, ask us to state, through the medium of our columns, that there are vacancies on board these ships for poor boys of good character who wish to go to sea. The vacancies arise because so many lads have recently been sent into the Royal Navy and mercantile marine. Mission workers and school teachers, as well as the clergy and ministers of all denominations, may be thankful to hear of these openings for aspiring young sailors of good character, and are particularly asked to note that the *Arethusa* and *Chichester* are *not* reformatories. The ages should be between fourteen and fifteen and a-half.

THE State Children's Aid Association has issued a letter to the chairman and members of the Children's Committee of the Metropolitan Asylums Board congratulating them on their determination to provide for the continuance of the education of the children while inmates of the houses, and also on the limited numbers they propose to accommodate in their houses for feeble-minded children. They also approve of the Homes for Convalescents being limited to receive 25, but think large institutions for the treatment of ophthalmia cases inexpedient. They point out that, as children become more scattered, ophthalmia may become as rare among pauper children as among those of other classes, and quote the valuable fact brought out in Dr. Stephenson's report that among normal children only 0·46 per cent. had active trachoma, whereas 5·72 per cent. of those in the London pauper schools were found to be similarly affected. The association is also of opinion that the Children's Committee of the Metropolitan Asylums Board should obtain compulsory powers to inspect periodically the children in the existing metropolitan schools, workhouses, and infirmaries in order to transfer to their care all children to whom the order of the Local Government Board extends. The letter concludes by suggesting that each home should be placed under the supervision of local managers whose sympathy and friendship would be valuable to the children and encouraging to the officials.

